# A Longitudinal Study of DMFT Counts in a Population of Ljubljana Over a Thirty Year Period

**DOI:** 10.3290/j.ohpd.a45072

**Published:** 2020-09-04

**Authors:** Eva Skaleric, Aleš Fidler, Uroš Skaleric

**Affiliations:** a Teaching Assistant, Department of Oral Medicine and Periodontology, University Clinical Center Ljubljana and Medical Faculty of Ljubljana, Ljubljana, Slovenia. Research; wrote the paper.; b Associate Professor, Department of Endodontics, University Clinical Center Ljubljana and Medical Faculty of Ljubljana, Ljubljana, Slovenia. Statistical analysis; proofread the paper.; c Academic Professor, Department of Oral Medicine and Periodontology, University Clinical Center Ljubljana, Ljubljana, Slovenia. Proofread the paper.

**Keywords:** epidemiological study, teeth, DMFT counts, subjects

## Abstract

**Purpose:**

Few longitudinal studies on changes of decayed, missing, or filled teeth (DMFT) counts in a population have been reported. This study aimed to evaluate the changes in DMFT counts in Ljubljana citizens in a 30-year period.

**Materials and Methods:**

238 dentate subjects that attended the third epidemiological study were invited. Ninety 45–95-year-old subjects (37.8%) responded to our invitation. Two (2.2%) edentulous subjects were excluded. Among the dentate subjects there were 28 men and 60 women. They were divided in six age groups with mean age of 45, 55, 65, 75, 85, and 95 years. The number of subjects in each age group was: 29, 12, 21, 18, 7, and 1, respectively. For evaluation of the state of teeth we used the DMFT index.

**Results:**

The average DMFT value for 45–95-year-old population was 19.3. Mean DMFT counts in all comparable age groups (45-, 55- and 65-years) decreased in 30 years. In 45 year olds they decreased from 17.5 to 15.7, in 55-year-olds they decreased from 20.4 to 19.2, and in 65 year olds they decreased from 22.5 to 20.7. An increase of the average number of present teeth (for 6.4 teeth in average) from the first to the fourth study in subjects of the same age was seen.

**Conclusion:**

Dental health in Slovenia has improved in 30 years. Average DMFT counts in subjects of the same age (45-, 55- and 65-years) have decreased. Ljubljana citizens have less decayed, less missing and more filled teeth than 30 years ago.

Despite great improvements in oral health in many countries and possible applied preventive measures,^8,21^ dental caries still remains an important oral health problem. The prevalence of dental caries among adults is still very high, affecting 2.5 billion people worldwide, as measured in 2015.^13^ High decayed, missing or filled teeth (DMFT) values (ie, 14) were measured in most industrialised countries and some countries of Latin America whereas caries prevalence in developing countries (Africa and Asia) was lower.^21^ According to the World Health Organization (WHO) these differences are related to availability of simple sugars in diets, to fluoride exposure/use and to dental treatment.^5^ Various studies have shown also that people with lower education and lesser income have higher DMF values.^4,15,19^ This is due to lack of access to dental care and differences in care utilisation because of social and economic disparities.^4,10,18^ However, the prevalence of caries in industrialised countries has decreased^17^ and many studies have shown a greater number of retained teeth in the adult population,^8,9,12^ which is mainly correlated with a more directed use of fluorides.^21^

Vrbič et al^27^ measured the caries prevalence of Slovenian children and adults in three studies in the last century (years 1987, 1993 and 1998). They examined the following age groups: 6-year-old subjects, 12-year-old subjects, 15-year-old subjects, 18-year-old subjects, 35–44-year-old subjects and 65 year olds. Results of the studies showed a big improvement in a 12-year period as there was a big increase in the percentage of children and adolescents without carious lesions (from 6% to 12% in 12-year-old population). In the 12-year period, also a decrease of DMFT counts in all other age groups was seen: 12-year-old subjects from 5.1 to 1.8; 15-year-old subjects from 10.2 to 4.3; 18-year-old subjects from 12.9 to 7.0; 35–44-year-old subjects from 20.5 to 14.7; and 65-year-old subjects from 27.0 to 22.5. The improvement was correlated with the preventive programmes which were organised in elementary schools in that period and included controlled tooth brushing with a fluoride gel 16–18 times yearly, better oral hygiene, and preventive fissure sealing.^27^

In Ljubljana, the capital of Slovenia, three epidemiological studies evaluated the state of teeth and periodontal tissues of Ljubljana citizens. The first study was performed in the period 1983–1987,^26^ the second one 10 years later in the period 1993–1997,^14^ and the third one 20 years later in the period 2005–2007.

In the first study,^26^ 1692 subjects were examined. Eighty-three (83; 4.9%) subjects were edentulous and excluded from the study. Results on 1609 15–65-year-old dentate subjects showed DMFT values from 9.9 (15-year-old subjects) to 22.5 (65-year-old subjects).

A total of 1609 dentate subjects from the first study were invited to the second study.^14^ Out of 555 subjects who came to the examination, 538 25–75-year-old subjects were dentate and included in the study. Results of the second study showed that the average DMFT counts increased from the first to the second study from (16.9 vs 19.1) which was mainly due to ageing of the population. However, in the 10-year period in between the two studies, DMFT counts in most age groups decreased.

Twenty years after the first study, 538 dentate subjects that had attended the first two studies were invited for a third examination. 247 (45.9%) 45–95-year-old subjects responded and 238 were still dentate. The results of the third study showed an increase of DMFT counts with age from 14.8 in 35-year-old subjects to 25.0 in 85-year-old subjects. The average DMFT value for 45–95-year-old population was 19.6, which is higher than in the previous two studies (19.6 vs 19.1 vs 16.9). This is due to ageing of the population and to an increase of the number of filled teeth.

The comparison of subjects of the same age in the three studies showed a decrease in the average number of missing teeth and an increase in the average number of filled teeth.

Due to a lack of longitudinal studies, we decided to perform a fourth epidemiological study on the state of the teeth and periodontal tissues^25^ of Ljubljana citizens who had already attended the first, the second and the third epidemiological study.

The aim of the study was to investigate the changes in DMFT counts and the number of teeth in the 30-year period in-between the first and the fourth study.

## Materials and Methods

This study was reviewed and approved by the National Medical Ethics Committee of the Republic of Slovenia (reference number: 25k/03/09).

We performed our study on Ljubljana citizens between the years 2016 and 2017 in the Department of Oral Diseases and Periodontology in Ljubljana University Dental Clinic. We invited all the 238 dentate subjects that had attended the third epidemiological study to a clinical examination of teeth and periodontal tissues. Finally, ninety 45–95-year-old subjects (37.8%) responded to our invitation and attended our study. Two (2.2%) subjects were edentulous and excluded from the study. Among the dentate subjects there were 28 men and 60 women. The number of subjects in each age group was as follows: 45 years = 29; 55 years = 12; 65 years = 21; 75 years = 18; 85 years = 7; and 95 years = 1.

For evaluation of the state of teeth of Ljubljana citizens we used the DMFT index according to criteria recommended by WHO,^28^ as the same index had been used in the first three epidemiological studies on the same population. According to the DMFT index, all the teeth that had decay received the letter D, all the teeth that were missing received the letter M and all the teeth that were filled or treated and had no decay received the letter F. DMFT counts in our study were examined on 28 teeth. Third molars were excluded.

The clinical examination was performed using a mouth mirror, a dental probe and artificial lighting, and was carried out by two calibrated dentists at the Department of Oral Diseases and Periodontology.

## Results

Table 1 shows the characteristics of the Ljubljana citizens by gender and age.

**Table 1:** The characteristics of the Ljubljana population by gender and age

Age (years)	Gender	N (Number of subjects)	
		Dentate	Edentulous
45	F M Σ	19 10 29	– – –
55	F M Σ	7 5 12	– – –
65	F M Σ	14 7 21	– 1 1
75	F M Σ	13 5 18	1 – 1
85	F M Σ	6 1 7	– – –
95	F M Σ	0 1 1	– – –
45–95	F M Σ	59 29 88	1 1 2

Key: M = male; F = female; Σ = M+F.

Table 2 shows that the average values of DMFT counts increase with age from 15.7 in 45-year-old population to 26.0 in 95-year-old population. The average DMFT value for 45–95-year-old Ljubljana citizens amounts to 17.8 for men, 20.0 for women and 19.3 for all of the population. The average number of decayed teeth (D) was between 0.3 (45-, 55- and 65-year-old subjects) and 1.0 (95-year-old subjects). The highest percentage of subjects with decayed teeth was found in 95-year-old population (100%) and the lowest in 75-year-old population (16.7%). The average number of missing teeth increased with age from 1.2 in 45-year-old subjects and 20.0 in 95-year-old subjects. The highest percentage of subjects with missing teeth was found in the 85- and 95-year-old population (100%) and the lowest in the 45-year-old population (55.2%). The average number of filled teeth was between 5.0 in 95-year-old subjects to 15.0 in 55-year-old subjects. The highest percentage (100%) of subjects with filled teeth was found in all age groups.

**Table 2:** DMFT counts (M ± SD) in 88 subjects according to age and gender

Age	Gender	N	D			M			F			DMFT		
			M	SD	%	M	SD	%	M	SD	%	M	SD	%
45	M	11	0.2	0.4	18.2	1.3	1.5	54.5	13.5	4.9	100.0	15.0	5.0	100.0
	F	18	0.4	0.9	27.8	1.2	1.5	55.6	14.6	3.6	100.0	16.2	4.2	100.0
	Σ	29	0.3	0.8	24.1	1.2	1.5	55.2	14.2	4.1	100.0	15.7	4.4	100.0
55	M	4	0.5	0.6	50.0	3.8	2.7	100.0	16.0	3.6	100.0	20.0	3.8	100.0
	F	8	0.3	0.4	25.0	4.0	5.8	87.5	14.5	5.3	100.0	18.8	3.3	100.0
	Σ	12	0.3	0.5	33.3	3.9	4.8	91.7	15.0	4.9	100.0	19.2	3.4	100.0
65	M	7	0.1	0.4	14.3	5.6	4.6	100.0	9.7	3.1	100.0	15.4	4.6	100.0
	F	14	0.4	0.6	28.6	11.5	8.3	92.9	11.6	5.7	100.0	23.4	4.3	100.0
	Σ	21	0.3	0.5	23.8	9.5	7.8	95.2	10.9	4.9	100.0	20.7	6.3	100.0
75	M	5	1.0	2.2	20.0	10.0	10.1	80.0	11.6	8.6	100.0	22.6	4.1	100.0
	F	13	0.2	0.6	15.4	8.6	6.9	92.3	12.8	4.2	100.0	21.0	4.0	100.0
	Σ	18	0.4	1.2	16.7	9.0	7.7	89.9	12.5	5.5	100.0	21.4	3.9	100.0
85	M	1	2.0	0.0	100.0	10.0	0.0	100.0	12.0	0.0	100.0	24.0	0.0	100.0
	F	6	0.2	0.4	16.7	14.1	8.2	100.0	8.8	3.4	100.0	23.2	5.6	100.0
	Σ	7	0.4	0.8	28.6	13.5	7.6	100.0	9.3	3.3	100.0	23.3	3.1	100.0
95	M	1	1.0	0.0	100.0	20.0	0.0	100.0	5.0	0.0	100.0	26.0	0.0	100.0
	F	0	–	–	–	–	–	–	–	–	–	–	–	–
	Σ	1	1.0	0.0	100.0	20.0	0.0	100.0	5.0	0.0	100.0	26.0	0.0	100.0
45–95	M	29	0.5	1.0	27.6	5.1	6.3	79.3	12.2	5.4	100.0	17.8	6.0	100.0
	F	59	0.3	0.7	23.7	6.9	7.7	81.4	12.9	4.8	100.0	20.0	5.0	100.0
	Σ	88	0.4	0.8	25.0	6.4	7.1	80.7	12.7	4.9	100.0	19.3	5.4	100.0

Key: M = male; F = female; Σ = M+F; N = number of subjects; % = percentage of subjects; D = decayed tooth; M = missing tooth; F = filled tooth.

**Fig 1:**
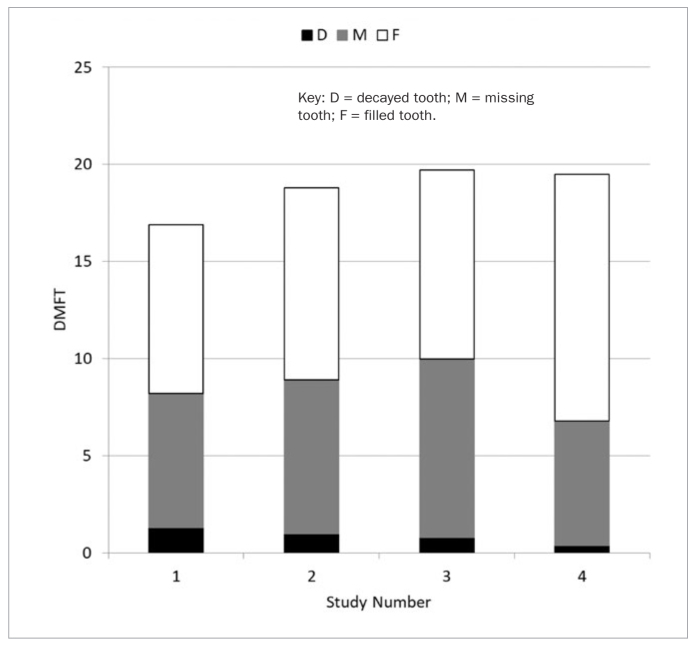
Mean DMFT counts in four epidemiological studies.

Figure 1 shows the comparison of distribution of the number of decayed, missing and filled teeth in all four studies. In 30 years, the average number of decayed teeth decreased from 1.3 to 0.4, the average number of missing teeth decreased from 6.9 to 6.4 and the average number of filled teeth increased from 8.7 to 12.7. The average DMFT value increased from 16.9 to 19.3 in a 30-year period.

**Fig 2:**
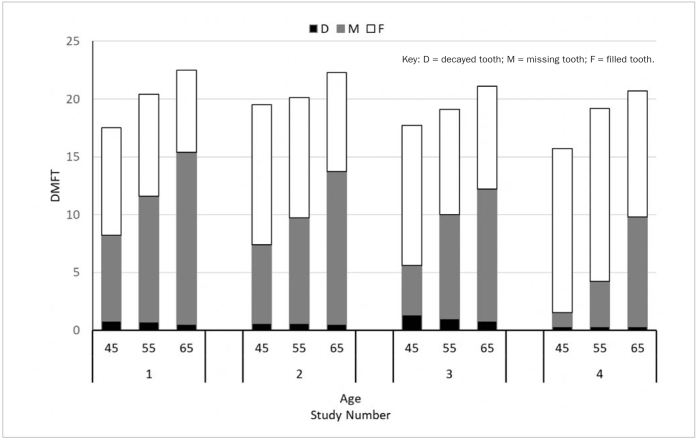
Mean D, M, F and DMFT counts for subjects of the same age (45-, 55- and 65-years) in four epidemiological studies.

Figure 2 shows the comparison of D, M, F and DMFT counts in 45-, 55- and 65-year-old subjects in between the four epidemiological studies. Mean DMFT counts decreased in all age groups in 30 years, which is mainly due to a decrease of average number of missing teeth in a 30-year period. In all age groups, the mean number of decayed and missing teeth decreased and the mean number of filled teeth increased in a 30-year period. In 45-year-old subjects DMFT decreased from 17.5 to 15.7, D decreased from 0.8 to 0.3, M decreased from 7.4 to 1.2, and F increased from 9.3 to 14.2. In 55-year-old subjects DMFT decreased from 20.4 to 19.2, D decreased from 0.7 to 0.3, M decreased from 10.9 to 3.9, and F increased from 8.8 to 15.0. In 65-year-old subjects, DMFT decreased from 22.5 to 20.7, D decreased from 0.5 to 0.3, M decreased from 14.9 to 9.5, and F increased from 7.1 to 10.9.

Table 3 shows the average number of teeth in all age groups in all four epidemiological studies. It is seen that in all four studies the number of present teeth in the oral cavity decreased with age. Table 3 also shows an increase of the average number of present teeth (for 6.4 teeth in average) from the first to the fourth study in all age groups that were examined in all four studies.

**Table 3:** Number of teeth (M ± SD) per person in four epidemiological studies.

Age (years)	Study 1 (N = 1609)		Study 2 (N = 538)		Study 3 N = 238)		Study 4 (N = 88)	
	M	SD	M	SD	M	SD	M	SD
15	27.5	0.9						
25	25.6	2.2	26.9	1.4				
35	22.7	3.7	24.7	3.5	26.8	1.4		
45	20.6	5.2	21.2	4.6	23.7	4.6	26.8	1.5
55	17.1	6.6	18.9	6.6	19.2	6.9	24.1	4.8
65	13.1	6.9	14.8	7.1	16.8	7.9	19.0	7.8
75			13.4	6.4	13.6	8.2	19.4	7.8
85					11.1	6.9	12.5	7.6
95							8.0	0.0

**Fig 3:**
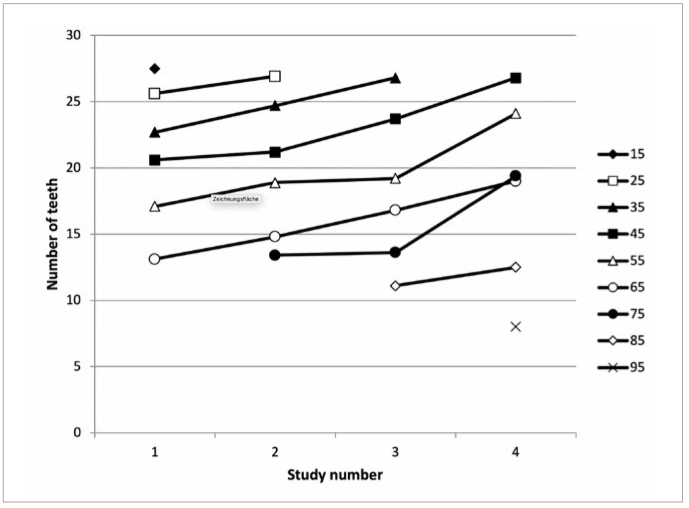
Mean number of teeth in all age groups in four epidemiological studies.

Figure 3 shows the increasing trends of the mean number of teeth from the first to the fourth study in all age groups that were examined in all four studies.

## Discussion

The results of the fourth epidemiological study on eighty-eight 45–95-year-old Ljubljana citizens showed that DMFT counts increase with age (from 15.7 in 45-year-old subjects to 26.0 in 95-year-old subjects) (Table 2). The same trend is seen also in the first three epidemiological studies on Ljubljana citizens.^14,26^ In a 30-year period DMFT counts in Ljubljana citizens have increased from 16.9 to 19.3, which is mainly due to an increase in the average number of filled teeth. With that we must consider that the subjects of the first study (15–65-year-old subjects) were 30 years younger than the subjects of the fourth study (45–95-year-old subjects).

If we compare the subjects of the same age (45-, 55-, and 65-year-old subjects) in all four Ljubljana epidemiological studies (Figure 2), the mean DMFT counts decreased in all age groups in 30 years, which is mainly due to a decrease of average number of missing teeth in a 30-year period. Subjects of the same age have less decayed, less missing and more filled teeth than 30 years ago.

The results of DMFT counts in our study on Ljubljana citizens are comparable to results that Vrbič^27^ found in his study on the adult population of Slovenia in the year 1998: 35–44-year-old subjects (14.7 vs 15.7) and 65-year-old subjects and older (22.5 vs 22.8). In the study of Vrbič,^26^ a decrease of DMFT counts in observed age groups in a 12-year period in between the three studies (years 1987, 1993 and 1998) is also seen. The improvement in the state of teeth (DMFT counts) in the citizens of Slovenia^27^ and Ljubljana^14,26^ at the end of the previous century was probably the consequence of the use of fluoridated toothpastes in Slovenia since the year 1985.^27^

In another study in Slovenia,^20^ the state of teeth and periodontal tissues of elderly living in nursing homes was investigated. The mean DMFT counts for subjects aged on average 79.8 years was 30.7, which is much greater than the average DMFT counts found in our 75–85-year-old subjects (22.5). That is probably due to the fact that Petelin et al^20^ included the third molars in their study for the difference of our study in which third molars were excluded.

Schiffner et al^24^ were investigating changes in the state of teeth and periodontal tissues in German children and adults in an 8-year period. They included the following age groups in their study: 12-year-old subjects, 15-year-old subjects, 35–44-year-old subjects and 65–74-year-old subjects. In adult subjects, a decrease of DMFT counts and a decrease in the average number of missing teeth was seen, which is comparable to the results obtained in our study. In all age groups, they also found a decrease in caries prevalence in an 8-year period.

Mariňo et al^16^ found comparable DMFT counts to the counts found in our study when investigating 354 sixty-year-old and older subjects in Chile. DMFT counts for the Chilean elderly were found to be 21.6, which is comparable to the DMFT counts we received for 75-year-old Ljubljana citizens (21.4).

Ahluwalia et al^1^ were observing the state of teeth and bone loss in the elderly that live in New York and regularly visit a dentist. The results of the study showed that these subjects have slightly lower DMFT counts than the elderly in our study (65–85-year-old subjects) (19.9 vs 21.8) which is probably due to the fact that the elderly in our study didn't all regularly visit a dentist.

In a study on the adult population of Valencia, Spain,^6^ also lower DMFT counts were found compared to those in our study. The average DMFT counts in 35–44-year-old subjects amount to 7.6 (45-year-old subjects in our study: 15.7) and in 65–74-year-old subjects 16.4 (65-year-old subjects in our study: 20.7; 75-year-old subjects: 21.4).

In a study in SE Sao Paolo, Brazil^22^ 1169 teachers 35–44 years old were examined. The DMFT index was investigated and the results obtained were not in accordance with the results obtained in 45-year-old subjects in our study: DMFT: 21.0 vs 15.7; D: 1.1 vs 0.3; M: 8.7 vs 1.2; F: 9.8 vs 14.2.

According to results of the first study on Ljubljana citizens,^26^ we can conclude that in a 30-year period in between the first and the fourth study, the mean number of decayed teeth decreased (1.3 vs 0.4), the mean number of missing teeth decreased (6.9 vs 6.4) and the mean number of filled teeth increased (8.7 vs 12.7).

Kalsbeek et al^11^ also found a decrease in the average number of decayed and missing teeth and an increase in average number of filled teeth in 30–54-year-old Dutch citizens in a 12-year period.

The results of our study also showed that the subjects of the same age have more teeth in their oral cavity than the subjects of the same age 30 years ago (Table 3). The trend towards maintaining natural teeth was also shown in some other studies.^8,9,11,23^

Hugoson et al^9^ analysed the caries prevalence and distribution in the age groups 20–80 years in 1973, 1983 and 1993. In the age groups 40–80 years, a steady increase in the number of teeth and an increasing number of decayed and filled tooth surfaces (DFS) was found in the 20-year period. A marked decrease in proximal DFS in 20–50-year-olds was also found. However, the results of this study are not completely comparable to the results of our study as Hugoson et al^9^ investigated each tooth surface for caries and our study investigated each tooth for caries. We are aware that our results are less precise in assessing caries prevalence when compared to the results obtained by Hugoson et al,^9^ but in our study DMFT index had to be utilised as it had been used in all three previous epidemiological studies performed on Ljubljana citizens. Other limitations of DMFT index are: the values do not provide any indication as to the number of teeth at risk or data that is useful in estimating treatment needs; the index gives equal weight to missing, untreated decay, or well-restored teeth; the index does not account for teeth lost for other reasons other than decay; the index does not account for sealed teeth.^2^

A disadvantage of our study is also the big dropout; however, despite this, we consider this study of importance as no similar study had ever been carried out. The advantage of this study is that it is longitudinal, and longitudinal studies on DMFT counts and caries prevalence are very rarely carried out in adult populations.^3^

## Conclusion

Dental health in Slovenia has improved.^7^

DMFT counts in all comparable age groups (45-, 55- and 65-year-old subjects) have decreased in a 30-year period. Ljubljana citizens have less decayed teeth and more maintained natural teeth in their oral cavity than Ljubljana citizens 30 years ago.

## References

[ref1] Ahluwalia KP, Cheng B, Josephs PK, Lalla E, Lamster IB (2010). Oral disease experience of older adults seeking oral health services. Gerodontology.

[ref2] Broadbent JM, Thomson WM (2005). For debate: problems with the DMF index pertinent to dental caries data analysis. Community Dent Oral Epidemiol.

[ref3] Carvalho JC, Schiffner U (2018). Dental caries in European adults and senior citizens 1996–2016: ORCA Saturday Afternoon Symposium in Greifswald, Germany – part II. Caries Res.

[ref4] Costa SM, Martins CC, Pinto MQC, Vasconcelos M, Abreu MHNG (2018). Socioeconomic factors and caries in people between 19 and 60 years of age: an update of a systematic review and meta-analysis of observational studies. Int J Environ Res Public Health.

[ref5] Edelstein BL (2006). The dental caries pandemic and disparities problem. BMC Oral Health.

[ref6] Eustaquio MV, Montiel JM, Almerich JM (2010). Oral health survey of the adult population of the Valencia region (Spain). Med Oral Patol Oral Cir Bucal.

[ref7] Glick M, Monteiro da Silva O, Seeberger GK, Xu T, Pucca G, Williams DM (2012). FDI Vision 2020: shaping the future of oral health. Int Dent J.

[ref8] Holst D, Schuller AA (2000). Oral health changes in an adult Norwegian population: a cohort analytical approach. Community Dent Oral Epidemiol.

[ref9] Hugoson A, Koch G, Slotte C, Bergendal T, Thorstensson B, Thorstensson H (2000). Caries prevalence and distribution in 20–80-year-old subjects in Jönköping, Sweden, in 1973, 1983, and 1993. Community Dent Oral Epidemiol.

[ref10] Ismail AI, Sohn W (2001). The impact of universal access to dental care on disparities in caries experience in children. J Am Dent Assoc.

[ref11] Kalsbeek H, Truin GJ, van Rossum GM, van Rijkom HM, Poorterman JH, Verrips GH (1998). Trends in caries prevalence in Dutch adults between 1983 and 1995. Caries Res.

[ref12] Kassebaum NJ, Bernabé E, Dahiya M, Bhandari B, Murray CJL, Marcenes W (2014). Global burden of severe tooth loss: a systematic review and meta-analysis. J Dent Res.

[ref13] Kassebaum NJ, Smith AGC, Bernabé E, Fleming TD, Reynolds AE, Vos T (2017). GBD 2015 Oral Health Collaborators. Global, regional, and national prevalence, incidence, and disability-adjusted life years for oral conditions for 195 countries, 1990–2015: a systematic analysis for the global burden of diseases, injuries, and risk factors. J Dent Res.

[ref14] Kovač-Kavčič M, Skalerič U (2001). The change of DMFT counts in Slovenia. Caries Res.

[ref15] Krustrup U, Holm-Pedersen P, Petersen PE, Lund R, Avlund K (2008). The overtime effect of social position on dental caries experience in a group of old-aged Danes born in 1914. J Public Health Dent.

[ref16] Mariño RJ, Cueto A, Badenier O, Acevedo R, Moya R (2011). Oral health status and inequalities among ambulant older adults living in central Chile. Community Dent Health.

[ref17] Marthaler TM (2004). Changes in dental caries 1953–2003. Caries Res.

[ref18] Newton JT, Bower EJ (2000). The social determinants of oral health: new approaches to conceptualizing and researching complex causal networks. Community Dent Oral Epidemiol.

[ref19] Paulander J, Axelsson P, Lindhe J (2003). Association between level of education and oral health status in 35-, 50-, 65-and 75- year-old subjects. J Clin Periodontol.

[ref20] Petelin M, Cotič J, Perkič K, Pavlič A (2012). Oral health of the elderly living in residential homes in Slovenia. Gerontology.

[ref21] Petersen PE, Bourgeois D, Ogawa H, Estupinan-Day S, Ndiaye C (2005). The global burden of oral diseases and risks to oral health. Bull World Health Organ.

[ref22] Rihs LB, da Silva DD, de Sousa Mda L (2009). Dental caries and tooth loss in adults in a Brazilian southeastern state. J Appl Oral Sci.

[ref23] Rozier RG, White BA, Slade GD (2017). Trends in oral diseases in the U.S. population. J Dent Educ.

[ref24] Schiffner U, Hoffmann T, Kerschbaum T, Micheelis W (2009). Oral health in German children, adolescents, adults and senior citizens in 2005. Community Dent Health.

[ref25] Skaleric E, Gaspirc B, Skaleric U (2019). A longitudinal study of periodontal treatment needs in a Ljubljana population over a 30-year period. Oral Health Prev Dent.

[ref26] Skalerič U, Kovač-Kavčič M (1991). DMFT counts in the adult population of Ljubljana, Yugoslavia. Community Dent Oral Epidemiol.

[ref27] Vrbic V (2000). Reasons for the caries decline in Slovenia. Community Dent Oral Epidemiol.

[ref28] World Health Organization (1997). Oral Health Surveys: Basic Methods.

